# Hemoglobin response to iron-folic acid supplementation and associated factors among anemic pregnant women attending the University of Gondar Comprehensive Specialized Hospital ANC ward Northwest, Ethiopia 2023: A longitudinal follow up study

**DOI:** 10.1371/journal.pone.0331599

**Published:** 2025-09-04

**Authors:** Asefu Fekadie, Bisrat Birke Teketelew, Melak Aynalem, Aregawi Yalew

**Affiliations:** Department of Hematology and Immunohematology, School of Biomedical and Laboratory Science, College of Medicine and Health Science, University of Gondar, Gondar, Ethiopia; Debre Markos University, ETHIOPIA

## Abstract

**Background:**

Anemia is the most frequent complication during pregnancy. Iron and folate deficiencies are the primary causes of anemia during pregnancy resulting from low hemoglobin concentration. Globally, preventive strategies such as iron and folic acid supplementation, improved dietary practice and deworming program play a crucial role in reducing the rate of anemia. Hemoglobin is improved as a result of iron-folic acid supplementation, though some factors affect good response. Due to limited studies to the study area, the main aim of this study was to assess the hemoglobin response to iron-folic acid supplementation and associated factors among anemic pregnant women. As iron and folic acid supplementation is the key intervention to reduce maternal and fetal complication, this study provides critical insights into the effectiveness of the intervention on improving hemoglobin level and can inform global maternal health strategies, particularly in resource limed settings where anemia remains a major public health concern.

**Methods:**

A longitudinal follow up study was conducted on a total of 357 anemic pregnant women at the University of Gondar Comprehensive Specialized Hospital, Northwest Ethiopia from June to October 2023. Simple random sampling technique was employed to select the study participants. Data were collected using pretested structured questionnaires. Hemoglobin was determined using *Sysmex kx21n hematological* analyze. Direct wet mount examination was performed to determine intestinal parasites. Moreover, serological tests were screened using rapid test kit immunochromatographic technique. Data was entered in to Epi-data version 4.6 software and exported to SPSS version 20 for analyses. Paired sample t-test was utilized to determine the change in hemoglobin. Both bi-variable and multivariable logistic regression were done to determine factors associated with the poor response of iron folic acid supplementation. A p-value of < 0.05 was considered as statistically significant.

**Results:**

The overall good hemoglobin response after IFA supplementation was 188/357 (52.7%). The median (IQR) of Hb was increased from 10.3 (9.6–10.49) gm/dL at baseline to 10.73 (9.78–12.0) gm/dL. Factors associated with poor hemoglobin response were residence (AOR = 2.4, 95% CI: 1.1 5.2), duration of IFA supplementation (AOR = 2.2,95% CI: 1.2, 3.7), intestinal parasite (AOR = 2.8, 95% CI: 1.3, 6.3), meat feeding habit less than two times per week (AOR = 1.7, 95% CI: 1.04, 2.9), green leaf vegetable and fruit feeding habit less than two times per week (AOR = 2.5,95% CI: 1.4, 4.0), coffee drinking habit (AOR = 1.9, 95% CI: 1.14, 3.3), parity (multiparous) (AOR = 2.9, 95% CI: 1.09, 7.2), HBsAg & HCV (AOR = 2.5, 95 CI: 1.15, 5.8) and stage of pregnancy (AOR = 4.0 95% CI (1.9,8.7). These factors in this study, showed significant association with poor hemoglobin responses.

**Conclusion:**

Hemoglobin level changed significantly from base line to end line. In this study, less than half of the study participants had poor hemoglobin response due to the aforementioned factors. Therefore, focused policies, health care facilitators and providers should strengthen efforts to provide information and create awareness about the benefits of iron and folic acid supplementation during pregnancy.

## Background

Iron and folic acid are essential nutrients for different biological activities. Folic acid is used for the synthesis of Deoxyribonucleic acid (abbreviated DNA) during blood cell production and is commonly used for the prevention and treatment of megaloblastic anemia. Iron is also an important nutrient required for many metabolic processes in the human body especially for the synthesis of Hemoglobin (Hb) and later for Red Blood Cell (RBC) production. Iron with folic acid supplements are given for the prevention and treatment of iron deficiency anemia (IDA) especially for pregnant women [1,2]. Most of the body’s iron is found in the RBCs in the form of Hb, which carries the oxygen delivered to the tissue [3]. Iron and folate deficiency during pregnancy are risk factors for poor neonatal health and increased infant mortality due to low birth weight, RBC disorders, and preterm delivery [4]. Iron deficiency, especially, is the major cause of low Hb concentration which results in moderate to severe anemia and subsequently contributes to various complications in both the fetus and pregnant women. [5]. The nutritional requirements and the development of the fetus are entirely dependent on the mother. The iron delivered to the fetus is from either maternal iron stores, absorption of iron from the maternal diet, or might be from the turnover of maternal erythrocytes [6].

During pregnancy, iron and folate deficiencies can result from one or the combination of the following factors including; abnormalities in utilization and metabolism, nutritional deficiencies, and increased demand for these nutrients [7]. There are absorption mechanisms of iron and folic acid from the diet, however, these are not sufficient for the individual requirements of daily iron and folic acid particularly for children and pregnant women. Hence, IFA supplementation is a necessary component of programs to control IDA and megaloblastic anemia including poor birth outcomes [8]. The World Health Organization (WHO) recommends all pregnant women receive IFAS regardless of their anemia status to reduce the risk of low birth weight, maternal anemia, iron deficiency, and neural tube defect [9]. After IFA supplementation, the Hb concentration of pregnant women improves and, pregnant women have a good response if the Hb concentration increases by 1g/dL after a minimum of one month of IFA supplementation [10].

The global prevalence of anemia related to pregnant women is around 36.8% [11], and 24.1% in the Americas, 52.5% in South East Asia, 25.1% in Europe, and the highest in Africa 61.3% [12,13]. In Ethiopia, the prevalence of anemia in pregnant women is 26.4% [14]. The most common cause of anemia in pregnancy is IDA which is due to some factors such as; increased need, increased loss, inadequate absorption, and in adequate utilization of nutrients [15]. Most of the anemia diagnosed during pregnancy is caused by IDA, followed by macrocytic anemia particularly megaloblastic anemia which is due to vitamin B12 and folate deficiency [16]. Increased folic acid requirement for fetal growth and inhibition of folate absorption by estrogen and progesterone during pregnancy are the major causes for folic acid deficiency [17]. The burden of IDA remains very high in pregnant women with global prevalence of 41.8% [18]. Despite WHO recommendation, the coverage of iron-folic supplementation in pregnant women remains very low in many countries. There is low adherence to IFA supplementation around Sub-Saharan countries with average coverage of 28.7% [19]. Some countries having low adherence, such as 1.4% in Burundi, and is better 73.0% in Senegal [20]. The adherence to IFA supplementation was 46.15% in Ethiopia [21].

According to studies conducted on the Hb response of pregnant women after IFA supplementation, good Hb response to IFAS was seen at 43.1% in Jordan [22], 39% in Tanzania [23], and 51.5% in Ethiopia [24]. As per the aforementioned studies, the Hb improvement in pregnancy after IFA supplementation was not sufficiently increased in all pregnant women as expected. This indicated that there are several factors may inhibit or hinder a good Hb response in pregnant women after IFA supplementation. These factors that may include; pre-existing factors such as chronic diseases, poor adherence, gestational issues, dietary factors, and some demographic factors [25]. Therefore, conducting this study aimed to address an important gap in understanding the outcome of IFA supplementation. It is also crucial for improving maternal and fetal health outcomes, guiding public health interventions, and informing policy decisions. Furthermore, identifying factors associated with the outcomes of IFA supplementation can help maintain protective factors and mitigate risk factors at an early stage.

## Methods and materials

### Study design, and period

A longitudinal follow up study was conducted from June to October 2023 at the University of Gondar Comprehensive and Specialized Hospital (UoG-CSH), Northwest Ethiopia, among anemic pregnant women after a minimum of one-month IFA supplementation to assess the Hb response to IFA supplementation.

### Study area and population

The study was conducted at the UoG-CSH, located in Gondar town, and the hospital serves over 7 million people in different wards including antenatal care, gynecology, and pediatrics. Annually, there were approximately 2200 anemic pregnant women attending UoG-CSH ANC ward. All anemic pregnant women who were attending the UoG-CSH ANC ward were considered as target population, and sampling was taken from all anemic pregnant women who were attending at the UoG-CSH ANC ward during the study period. Those who fulfilled the eligibility criteria, and who gave informed consent were included in this study. The study included all anemic pregnant women with Hb level of <11 g/dL and who were eligible to start IFA supplementation, and would have full adherence for IFA supplementation at the UoG-CSH during the study period. However, anemic pregnant women who had severe bleeding, recent transfusion, who had chronic kidney disease, who had taken erythropoietin therapy, and malaria positive were excluded from the study.

### Measurement variables

Hemoglobin response (good response and poor response) were considered as dependent variables for the study. Sociodemographic characteristics such as age, marital status, residence, maternal educational status, religion and occupation. Clinical characteristic such as presence of intestinal parasite infection, presence of chronic disease such as HBsAg and HCV, Human Immune Virus (HIV) infection, and presence of syphilis infection. Nutritional characteristic such as tea drink habit, coffee drink habit, meat feeding habit, green leaf vegetable and fruit feeding habit and daily feeding time. Obstetric factors such as parity, number of ANC visits, abortion, and stage of pregnancy in months, duration of IFA supplementation were independent variables for the study.

### Operational definitions

**Anemia;** According to WHO, pregnant women with Hb value of <11g/dl in the first and third trimester, < 10.5 g/dl in the second trimester are considered as anemic [26].

**Good response:** is the increment of hemoglobin value by at least 1g/dL after a minimum of one month IFA supplementation was considered as a good response [10].

**Poor** **response:** A change in hemoglobin value by less than 1g/dl after a minimum of one month IFA supplementation was classified as poor or inadequate response [27].

**Adherence;** was considered for a pregnant woman who took at least 65% of the expected dose of IFA supplementation in the previous week prior to the study, which is an equivalent of taking a single tablet daily for four days in the week consecutively or 20 tablets in a month without missing the prescribed dosage [28]. Whereas non Adherence was considered for a pregnant woman who took a single tablet daily less than four days in the week [29].

### Sample size determination and sampling technique

The sample size (n) was calculated using the single population proportion formula **as** (Z α/2)^2^ p (1-p)/d^2^. Where P = best estimate of population proportion from previous study, d = margin of error and Z = standard score corresponds to 1.96. Adequate Hb response based on the previous study conducted in Mekelle city was 51.5% [24]. For the calculation, a 95% confidence interval and a 5% margin of error was used to get a final sample size of 383.


$$\frac{{\left(Z\frac{\;a}{2}\right)}^{2}\;\;x\;P(1-P)}{d2}=\frac{\left(1.96{)}^{2}\;x\;0.515(1-0.515\right)}{(0.05{)}^{2}}=383$$

To minimize errors arising from the likelihood of lost flow up, 10% percent of the sample size (38) was added to get a final sample size which was 422. The study participants were then selected by simple random sampling technique.

### Data collection procedure

#### Socio-demographic and clinical data collection.

Socio-demographic characteristics of the study participants such as age, residence, occupation, educational status and nutritional characteristics were collected using a structured questionnaire via a face-to-face interview by trained clinical nurses. The questionnaires were developed by using the WHO guideline and further reviewing different literatures. Clinical characteristics of the study participants like hypertensive and diabetic mellitus, were collected by reviewing the patient’s medical record using a checklist. Participants’ statuses regarding hepatitis B and C viruses, syphilis, HIV, and intestinal parasites were determined through laboratory tests performed by skilled laboratory professionals.

The quality of socio-demographic and clinical data was ensured through the questionnaire, that was prepared in English and translated to Amharic then back to English to check consistency. Training was given to data collectors on the study’s purpose and significance, confidentiality, study participants’ rights, consenting, interview techniques, laboratory test procedures, and quality control before actual data collection, and close supervision was made during the data collection period. To standardize the questionnaire, a pretest was conducted on 5% of the sample size on randomly selected participants for its reliability and validity before actual data collection at Gebrael Health Center, Gondar Northwest Ethiopia. Socio-demographic, nutritional, and clinical characteristics were collected by trained clinical nurses under the supervision of the investigator. The laboratory tests were performed by medical laboratory technologists and experienced hematologists. Furthermore, investigators were closely followed up and frequently inspected the data collection processes to ensure the completeness, accuracy, clarity, and consistency of the data and gave timely feedback to the data collectors. The principal investigator also reviewed the collected data daily to ensure its completeness and accuracy.

#### Sample collection and laboratory method.

About 5 mL of venous blood sample was collected from the study participants by laboratory technologists. The sample was drawn after disinfecting the medial cubital vein in the forearm with a swab soaked in 70% alcohol solution. A 3 mL blood was transferred to ethylene di-amine tetra acetic acid (EDTA) test tube for complete blood count analysis. The standard operating procedures, daily maintenance, weekly maintenance, and internal quality control procedures for the analyzer were strictly adhered throughout the research process. The remaining 2 mL blood was transferred in to serum separator (gel) test tube for screening of HBsAg, HCV, syphilis and HIV before the initiation of IFA supplementation. The second 3 mL of blood sample was collected after one-month duration of IFA supplementation for the second Hb determination from the same study participants and who took four and more tablets per week. The Hb value before and after IFA supplementation was determined using the automated hematological analyzer*, (Sysmex kx-21, Japan)*. Screening tests for HIV, hepatitis B virus, hepatitis C virus and syphilis were conducted using kit based immunochromatographic techniques following standard procedure and algorithm. The reliability of the test results were ensured with known positive and negative samples for each test category. Approximately 1 gram of stool sample was collected and examined within 30 minutes to 2 hours. Delayed samples were preserved in 10% formalin and examined at a later time.

### Data processing and analysis

The data were collected, coded, and entered into Epi data version 4.6, then transferred to and analyzed by SPSS version 20 software. Descriptive statistics were used to summarize the characteristics of the study participants. Shapiro–Wilk test was utilized to check the normality of data. Wilcoxon signed-rank test was utilized to estimate the changes in median values of Hb between baseline and end point following IFA supplementation. The bivariable and multivariable logistic regression models were employed to identify factors associated with poor Hb response after IFA supplementation. Hosmer-Lemeshow was performed to check the model goodness-of-fit, and with included independent variables, the final model was well fitted. Crude odds ratio (COR) and adjusted odds ratio (AOR) with the corresponding 95% CI were calculated to show the strength of the association. Finally, in the multivariable analysis, variables with a p-value less than 0.05 were considered as statistically significant.

#### Ethical consideration.

The study was conducted after the proposal was reviewed and approved by the Research and Ethical Review Committee of the School of Biomedical and Laboratory Sciences, Colleges of Medicine and Health Sciences, University of Gondar. Then the ethical clearance was obtained and submitted to the UoG-CSH. Then, a permission letter was obtained from UoG-CSH Chief Clinical Director. The data was collected after written consent was obtained from each study participant. The objective of the research was explained to the study participants. Participation in the study was on a voluntary basis, and refusal was possible. To ensure the confidentiality of the data, study participants were identified with codes, and only authorized persons had accessed the data. Study participants with critical or panic results were reported to the physicians for the management and treatment.

## Results

### Socio-demographic and clinical characteristics

A longitudinal follow up study was conducted on a total of 357 individual with 85% response rate. The median (IQR) age of study participants was 32 (28–36) years. Majority of the study participants were between 26 and 35 years 204 (57.1%). Most of the participants were from urban area 249 (69.7%), and more than half of participants were married 243 (68.1%) (Table 1).

**Table 1:** Sociodemographic characteristics of study participants at the UGCSH, Northwest Ethiopia, 2023 (N = 357).

Variables	Categories	Frequency (n)	Percentage (%)
Age categories in year	18-25 years	45	12.6
	26-35 years	204	57.1
	36-44 years	108	30.3
Residence	Urban	249	69.7
	Rural	108	30.3
Educational status	No formal education	59	16.5
	Primary school	50	14.0
	Secondary school	89	24.9
	College& University	159	44.5
Marital status	Single	38	10.6
	Married	243	68.1
	Widow	46	12.9
	Divorced	30	8.4
Occupation	Employed	124	34.7
	Daily laborer	43	12.0
	Merchant	90	25.2
	Farmer	57	16.0
	Housewife & others**	43	12.0

Note: others** Occupation No work, Median (IQR) age of study participants was 32 (28–36) years.

### Obstetric factors, clinical characteristics and nutritional characteristics of the study participants

About 158 (44.3%) of the study participants were in the second trimester, and 311 (87.1%) were primiparous. 123 (34.1%) of the study participants had history of abortion in their previous pregnancy. From a total of study participants 48 (13.4%) were infected with intestinal parasites, predominantly 29/48 (60.4%) *A.lumbricoides. 159 (*44.5%) of the participants had coffee drinking habit, and 81 (22.7%) had no time preference to drink coffee. Around half 183 (51.3%) of the participants ate meat greater than two times per week (Table 2).

**Table 2:** Obstetric factors, clinical, and nutritional characteristics of study participants (N = 357).

Variables	Categories	Frequency (n)	Percentage (%)
Duration of IFAS	4-5 weeks	173	48.5
	>6 week	184	51.5
Frequency of ANC visit	≤ 3 times	188	52.7
	≥ 4 times	169	47.3
History of Abortion	Yes	123	34.5
	No	234	65.5
Stage of pregnancy	First trimester	82	23.0
	Second trimester	158	44.3
	Third trimester	117	32.8
Parity	Primiparous	311	87.1
	Multiparous	46	12.9
Presence of chronic disease	Yes	46	12.9
	No	311	87.1
Presence of HBsAg &HCV infection	Positive	47	13.2
	Negative	310	86.8
Presence of HIV infection	Positive	13	3.6
	Negative	344	96.4
Presence of syphilis infection	Positive	18	5.0
	Negative	339	95
medication taken in last two weeks	Yes No	76 281	21.3 78.7
Types of medication taken	Antibody	13	21.6
	Antibiotics	18	23.7
	Aspirin -Insulin	33	43.4
	Anti-parasitic	14	18.3
Intestinal parasite infection	Present	48	13.4
	Absent	309	86.6
Types of Intestinal parasite	*A.lumbricodes* *G. lambilia* *Hookworm* *E.histololtica*	29 8 6 5	60.4 16.6 12.5 10.5
Meat feeding habit	2 times/week	174	48.7
	≥ 2 times/week	183	51.3
Green leaf vegetable & fruit feeding habit	<2 times/week ≥ 2 times/week	111 246	31.1 68.9
Coffee drinking habit	Yes	159	44.5
	No	198	55.5
Time of coffee drinking	Before meals After meals No time preference	5 73 81	1.4 20.4 22.7
Tea drinking habit	Yes No	112 245	31.4 68.6
Time of tea drinking	Before meals After meals No time preference	6 91 15	1.7 25.5 4.2

Note: HBsAg: Hepatitis B surface antigen, HCV: Hepatitis C virus, HIV: Human Immune Virus.

### Hemoglobin response to IFA supplementation among anemic pregnant women at the UGCSH, 2023 (N = 357)

**Fig 1:**
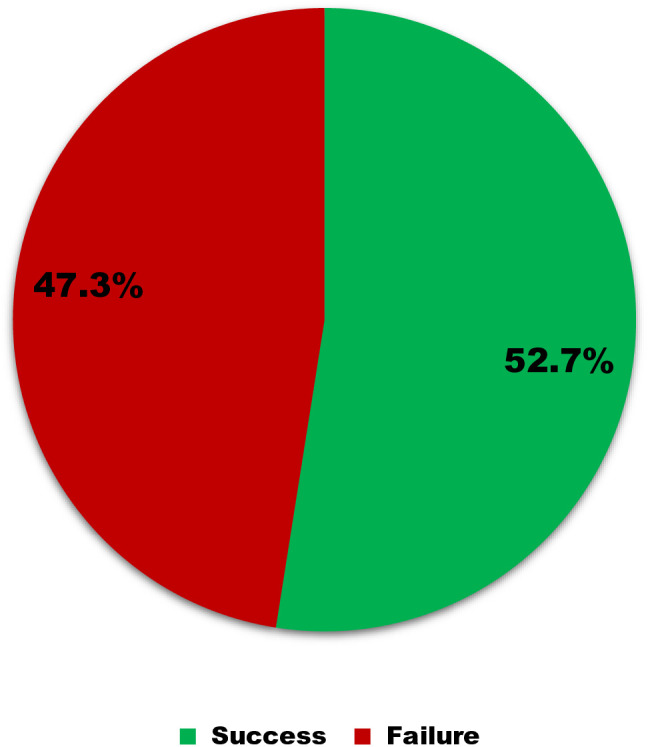
Outcome indicators of the Hb response of IFA supplementation among anemic pregnant women attending the University of Gondar Comprehensive Specialized Hospital Antenatal Care ward, Northwest, Ethiopia 2023. Good response = 188 (52.7%) and poor response = 169 (47.3%).

Hemoglobin response was measured in pregnant women with Hb value less than 11 g/dL. The median (IQR) of Hb was increased from 10.3 (9.6–10.49) gm/dL at baseline to 10.73 (9.78–12.0) gm/dL. In this study the overall good Hb response to IFA supplementation was 188(52.7%) (Fig 1).

### Hemoglobin response to IFA supplementation among anemic pregnant with co-morbidity at UGCSH, 2023 (n = 124)

**Fig 2:**
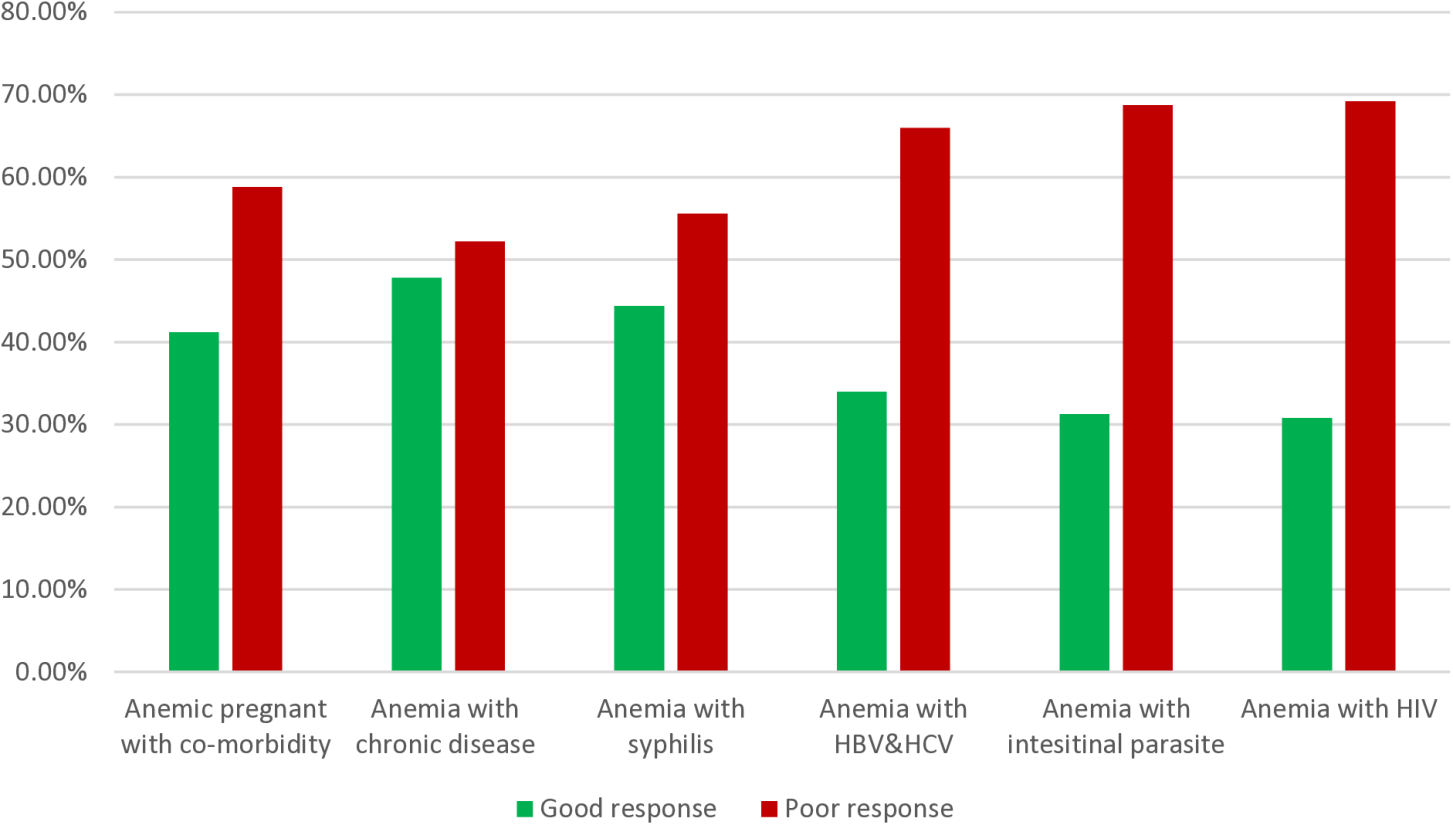
Hemoglobin response to IFA supplementation among anemic pregnant women with co-morbidity attending at the University of Gondar Comprehensive Specialized Hospital Antenatal Care ward, Northwest, Ethiopia 2023.

In this study, poor Hb response to IFA supplementation among anemic pregnant women with co-morbidity was high 58.8%. Particularly poor Hb response among anemia with HIV was 69.2% (Fig 2).

*Note*; *chronic disease* included diabetes mellitus, hypertensive and heart disease

### Factors associated with poor hemoglobin response after IFA supplementation among anemic pregnant women

Both bivariable and multivariable logistic regression analysis was done to identify factors associated with poor hemoglobin response to IFA supplementation. In the bivariable analysis, variables including, age category, residence, marital status, education level, occupation, religion, green leaf vegetable and fruit feeding habit, duration of IFA supplementation, intestinal parasite infection, presence of HBsAg & HCV, presence of chronic disease, coffee drinking habit, parity, stage of pregnancy, history of abortion, frequency of ANC visit, medication taken for last two weeks, meat feeding habit, tea drinking habit and medication taken for last two weeks. Then a variable with P-value ≤ 0.25 in the bivariable analysis were entered in in multivariable logistic regression analysis, and variables including age category, residence, marital status, education status, occupation, green leaf vegetable and fruit feeding habit, duration of IFA supplementation, intestinal parasite infection, presence of HBsAg & HCV, parity, stage of pregnancy, meat feeding habit, medication taken for last two weeks and coffee drinking habit were candidates for multiple logistic regression analysis. After analysis, variables which were significantly associated with poor Hb response to IFA supplementation include rural residence (AOR = 2.4,95% CI:1.15,5.2, p = 0.021), less than 5 weeks duration of IFA supplementation (AOR = 2.2, 95% CI:1.2,3.7, p = 0.004), intestinal parasite infection (AOR = 2.8,95% CI:1.3,6.3, p = 0.009), meat feeding habit less than two times per week (AOR = 1.7,95% CI:1.04,2.9, p = 0.035), green leaf vegetable and fruit feeding habit less than two times per week (AOR = 2.5,95% CI:1.4,4.0, p = 0.001), coffee drinking habit AOR = 1.9, 95% CI (CI:1.14,3.3, p = 0.015), parity (multiparous) (AOR = 2.9,95% CI:1.09,7.2, p = 0.019), stage of pregnancy (AOR = 4.0,95% CI:1.9,8.7, p < 0.001) and HBsAg & HCV positive (AOR = 2.5,95%:1.15,5.8, 0.022) were statistically significant associated with poor Hb response (Table 3).

**Table 3:** Factor associated with poor hemoglobin responses to IFA supplementation among anemic pregnant women attending at the UGSH ANC ward, Northwest, Ethiopia 2023 N = 357.

Variables	Categories	Good response n (%)	Poor response n (%)	COR (95%CI)	AOR (95%CI)	p-value
Age categories	18-25	29 (64.44)	16 (35.56)	1^a^	1^a^	1^a^
	26-35	107 (2.45)	97 (47.55)	1.6(0.8-3.2)	1.8(0.7-4.2)	0.160
	36-44	51 (47.22)	57 (52.78)	2.0(0.9-4.1)	1.6(0.6-4.3)	0.200
Residence	Urban	153 (61.44)	96 (38.56)	1^a^	1^a^	1^a^
	Rural	34 (31.48)	74 (68.2)	3.4(2.1-5.6)	2.4(1.1-5.2)	0.021*
Educational status	College& university	93 (58.49)	66 (41.50)	1^a^	1^a^	1^a^
	Primary	24 (48.0)	26 (2.0)	1.5(0.8-2.8)	0.8(0.3-2.2)	0.700
	School					
	Secondary school	55 (61.79)	34 (38.20)	0.8(0.5-1.4)	0.7(0.3-1.6)	0.400
	No formal education	15 (25.40)	44(74.56)	4.1(2.1-8.04)	1.4(0.5-3.9)	0.400
Marital status	Single	23 (60.52)	15 (39.48)	1^a^	1^a^	1^a^
	Married	122 (50.20)	121 (49.8)	1.5(0.7-3.0)	1.0(0.4-2.3)	0.900
	Divorced	28 (60.86)	18 (39.14)	0.9(0.4-2.3)	0.4(0.15-1.4)	0.170
	Widowed	14 (38.88)	16 (61.12)	1.7(0.6-4.6)	1.09(0.3-3.7)	0.800
Occupation	Employed	78 (62.9)	46 (37.1)	1^a^	1^a^	1^a^
	Daily laborer	21 (48.83)	22 (51.17)	1.7(0.8-3.5)	1.5(0.5-4.2)	0.400
	Merchant	51 (56.66)	39 (43.34)	1.2(0.7-2.2)	1.2(0.5-2.8)	0.500
	Farmer	17 (29.82)	40 ((70.18)	3.9(2.0-7.8)	1.7(0.5-5.1)	0.300
	Housewife& others	20 (46.51)	23 (53.49)	1.9(0.9-3.9)	1.1(0.4-3.0)	0.700
Duration of IFAS	>5 weeks4	110 (59.78)	74 (40.22)	1^a^	1^a^	1^a^
	−5 weeks	77 (44.5)	96 (55.5)	1.8(1.2-2.8)	2.2(1.2-3.7)	0.004*
Stage of pregnancy	First trimester	60 (73.17)	22 (26.82)	1^a^	1^a^	1^a^
	Second trimester	77 (48.73)	81 (51.26)	0.3(0.2-0.6)	3.4(1.7-7.1)	0.001*
	Third trimester	50 (42.73)	67 (57.26)	0.4(0.2-0.9)	4.0(1.9-8.7)	<0.001*
Parity	Primiparous	175 (56.27)	136 43.73)	1^a^	1^a^	1^a^
	Multiparous	12 (26.08)	34 (73.920	3.6(1.8-7.3)	2.9(1.19-7.2)	0.019*
Presence of HBsAg &HCV infection	Negative Positive	171(54.98) 16 (34.04)	139(45.02) 31(65.95)	1^a^ 2.3(1.2-4.5)	1^a^ 2.5(1.15-5.8)	1^a^ 0.022*
Medication taken for last two weeks	No	154 (55.19)	125 (44.80)	1^a^	1^a^	1^a^
	Yes	33 (42.30)	45(57.69)	1.6(1.0-2.7)	1.6(0.8-3.1)	0.115
Intestinal parasite infection	Absent Present	172 (55.66) 15 (31.25)	137 (44.34) 33 (68.75)	1^a^ 2.7(1.4-5.2)	1^a^2.8(1.3–6.3)	1^a^0.009*
Meat feeding habit	≥ 2x/week	109 (59.56)	74 (40.43)	1^a^	1^a^	1^a^
	Never& < 2x/week	78 (44.82)	74 (40.43)	1.3(0.8-2.0)	1.7(1.04-2.9)	0.035*
Green leaf vegetable & fruit feeding habit	≥ 2x/week	143 (58.13)	103 (41.86)	1^a^	1^a^	1^a^
	Never& < 2x/week	44 (39.63)	67 (60.36)	2.1(1.3-3.3	2.5(1.4-4.5)	0.001*
Coffee drinking habit	No Yes	125 (63.13) 62 (38.99)	73 (36.86) 97 (61.00)	1^a^ 2.6(1.7-4.1)	1^a^ 1.9(1.14-3.3)	1^a^ 0.015*

***Note: 1***^***a***^ *indicates reference category,* *Indicates statistically significant at a level of P < 0.05. ***AOR:*** *Adjusted odd ratio,* ***COR:*** *Crude odd ratio,* ***CI:*** *Confidence interval.* ***Parity***; ***primiparous****; a woman who has bearing the first offspring,* ***multiparous*** *a woman who has given birth two or more times. The p- value in indicated for AOR.*

## Discussion

Anemia is a prevalent health issue during pregnancy, primarily caused by iron and folic acid deficiencies [30]. The increased physiological demand for iron and folate during pregnancy often exceeds dietary intake, making it difficult to meet these nutritional requirements through diet alone [31]. Consequently, routine iron and folic acid (IFA) supplementation is recommended, particularly in resource-limited settings [32]. Evidence from previous studies has consistently demonstrated that iron supplementation with or without folic acid, significantly reduces the incidence of anemia during pregnancy [33,34]. In this study, the outcomes of IFA supplementation were evaluated and factors associated with poor Hb response among anemic pregnant women were identified.

The result of this study revealed that the overall good Hb response to IFA supplementation was 52.7%. This is higher than the studies conducted in Kenya [35], Tanzania 39% [23], Australia, 28.4% [36], Roma 21.2% [37], Maulana Azad Medical College 30% [38], and Jordan 43.1%) [22]. The discrepancy in reporting might be due to geographical variation, differences in socioeconomic status, and dietary habits of the study participants, and the use of a large sample size in this study [39].

The outcome of the current finding is in line with a study conducted in Mekelle 51.5% [24], and Pakistan 49% [40]. This agreement may be due to similarities in demographic, socioeconomic, or cultural characteristics among the populations in these regions. The other possible reason for the agreement is similarities in methodologies such as study design, data collection tool and analysis method. Based on this study’s finding, there is notably good Hb response in pregnant women after IFA supplementation. Effective coverage of antenatal iron and folic acid (IFA) supplementation is important to prevent adverse maternal and newborn health outcomes [41]. As per WHO recommendation of universal daily iron supplementation in pregnant women, antenatal care intervention should include multiple micronutrient supplements including iron and folic acid [42]. In this finding, poor Hb response among anemic pregnant women with co-morbidity was 58.8%. This result showed that more poor response among anemic pregnant woman with co-morbidity rather than anemic pregnant woman without co-morbidity. Severity of anemia would increase with the presence of other medical comorbidities. This was due to different factors like treatment effect, nutritional deficiency, weak immunity (immune deficiency), decreased RBC production, increased RBC distraction, and it may be also infective RBC production, and iron deficiency from chronic blood loss among anemia with co-morbidity rather than anemic pregnant without co-morbidity [43,44].

In this study, the overall poor Hb response to IFA supplementation among anemic pregnant women was 47.3%.There were different factors assessed for poor Hb response, including; the participants were from rural area, parity (multiparous), intestinal parasite infection, duration of IFA supplementation less than five month, meat and vegetable consumption less than two times in a week, HBV&HCV positive, stage of pregnancy and coffee drinking habit were associated with poor hemoglobin response to IFA supplementation. Participants from rural resident were 2.4 times more likely to experience failure compared to those from urban. The possible reason might be more participants were from rural area, no formal education, divorced, widowed, low ANC visit, the other reason may be the distance of facility, awareness about benefits of IFA supplementation, availability of supplements [45].

Parity were also another factor significantly associated with poor response; multiparous anemic pregnant women had 2.9 times failure of IFA response when compared to primiparous anemic pregnant women. This is because women might not have enough time to restore depleted nutritional stores before the onset of the next reproductive cycle when there are short gaps between pregnancies [25]. Another explanation could be that every pregnancy raises the risk of bleeding before, during, and after delivery. This could lead to a decrease in the hemoglobin’s first response to IFA supplementation in multiparous pregnancies [46].

Duration of IFA supplementation was also another factor for poor Hb response to IFA supplementation. Anemic pregnant women who took IFA less than five weeks had 2.2 times poorer response than anemic pregnant women who took IFA more than five weeks. This is due to the shorter time of IFA the lower response to Hb, because it is better to take IFA for longer time to meet the three months life span of RBC to prevent anemia [47]. The WHO has recommended a six-month regimen of a daily supplement containing 60 mg of elemental iron with 400 *μ*g folic acid, which is supplied to pregnant mothers during the first trimester or as soon as possible. Later on and it is provided when pregnant mothers come for antenatal care [48]. Accordingly, in Ethiopia, the national guideline for control and prevention of micronutrient deficiency also recommends daily iron folic acid supplementation for 180 days during pregnancy or it could be finished after delivery if the mother did not finish the full dose during pregnancy [49].

Participants who were third trimesters had 4.0 times failure of IFA response than first and second trimester. This was due to iron requirements are not uniform throughout the 3 trimesters of pregnancy. In the first trimester, the requirements (estimated at ∼0.8 mg/d) are lower than before pregnancy as menstruation stops. As pregnancy advances, maternal RBC mass increases and placental and fetal growth accelerates, which result in the rise in physiologic iron requirements to 3.0–7.5 mg/d in the third trimester [50]. Additionally during pregnancy, hemodilution leads to a reduced Hb concentration and iron requirements begin to increase and continue to do so throughout the third trimester of pregnancy [51].

Participants who had intestinal parasite were 2.8 times failure of IFA response than participants who had no intestinal parasite. This finding is consistent with other study in Peshawar [52]. On microscopic stool examination, it was found that, *A. lumbricoides* (8.1%), *G. labia* (3.6%) and *Hookworms* (1.7%). The helminthic infection like *A. lumbricoides* and *Hookworm* causes anemia by reducing iron uptake from the intestine, directly sucking blood, and interfering directly and indirectly in iron metabolism, as the number of Ascaris eggs increase in the body of infested person, they will suffer from decreasing Hb level [53]. Parasites like *G. labia* causes anemia by destructing the intestine mucosal structure, that influences in micronutrients absorption, storage, and utilization such as iron. All these mechanisms also affect hosts’ nutritional status by reducing appetite, competing for iron and causing diarrhea/dysentery and decreased iron absorption and then alter their immune system [54,55].

Participants who were HBsAg and HCV positive were 2.5 times failure of IFA response than participants who had no HBsAg &HCV. This was due to destruction of red blood cells before their normal 120-day life span by direct viral action which results hemoglobinuria derived from free Hb, erythrocyte fragility due to abnormal globulins, and increased hemolytic toxicants by decreased hepatic clearance, rapid splenomegaly and decreased Hb concentration [56,57]. The other basic reason is, chronic liver infection like hepatitis has an impact on alteration of iron metabolism by producing special protein which regulates ion metabolism such as hepcidin. During liver infection the hepatocytes produces the protein hepcidin and blocks iron absorption even though in low body iron concentration. Therefore, the high onset of poor Hb response for IFA supplementation in hepatitis infected pregnant women might be due to the hepcidin activity [58]. Although no statistical significance was observed, co-morbidities particularly in pregnant women living with HIV showed a high treatment failure rate, with 69.2% experiencing poor Hb responses. This finding highlights the need for special attention and tailored interventions for pregnant women with HIV to mitigate the impact of the disease and reduce the associated risk of anemia. Pregnant women living with HIV are at high risk for micronutrient deficiency particularly iron [59]. HIV-related abnormal erythropoiesis and impaired hemoglobin synthesis may be further exacerbated by antiviral agents such as Zidovudine. Supporting this, studies have shown that hemoglobin recovery is slower following oral iron supplementation in this group [60,61]. These findings emphasize the importance of developing specific intervention plans, including optimized iron supplementation strategies, to address the effects of the virus and antiviral treatments on hemoglobin recovery in pregnant women living with HIV.

Consumption of meat is another factor which showed significant association with poor hemoglobin responses in this study. Pregnant women who ate meat less than two times per week were 1.7 times more likely to have poor response than who ate more than two times per week. This finding is supported by another studies conducted in Pakistan [30], and Mekelle town [25], in which pregnant women who ate meat two or more times per week had higher mean Hb concentrations [30]. The possible reason for this might due to the heme iron found in animal source like meat has fast absorption rate than non-heme iron [62]. Study participants who ate green leafy vegetables & fruit less than two times per week were 2.5 times more likely to failure of IFA response than pregnant mothers who ate green leafy vegetables & fruit more than two times per week. This finding was supported by previous studies conducted in Pakistan and Turkey [30,63]. The possible reason could be the presence of ascorbic acid in fresh fruits like lemon, orange can increases the absorption of non heme iron [64], by creating a chelate with ferric iron (Fe^3+)^ at a stomach acidic pH which stays soluble at the alkaline pH of the duodenum [65].

Coffee drinking habit also was also another factor which showed significant association with poor hemoglobin responses. Pregnant women who drank coffee were 1.9 times failure of IFA response than pregnant women who not drink coffee. This finding was supported by the study in Korea [66]. This is due to naturally occurring compound found in coffee called polyphenols, which are known to inhibit the absorption of non-heme iron by binding to iron and form insoluble complexes in the intestines, making it difficult for the body to absorb the iron [67]. This study has both strengths and limitations. One of its key strengths is the large sample size, which enhances the reliability of the findings. Additionally, analyzing hemoglobin response following IFA supplementation and identifying associated factors serves as a valuable contribution by raising awareness and highlighting factors linked to poor responses to IFA supplementation. However, the study has certain limitations. Some variables were based on recall knowledge, which may introduce recall bias. The study also lacked the ability to include laboratory methods, such as serum iron profile assessments. Furthermore, it did not classify types of anemia or assess micronutrient levels, which could have helped identify key contributors to anemia in pregnant women. Future research should address these limitations by incorporating micronutrient assessments and exploring the broader causes of anemia to provide a more comprehensive understanding.

## Conclusion

In this study, the evaluation of Hb levels before and after IFA supplementation showed a significantly higher level at the end line compared to the baseline. According to the result of this study, the overall good Hb response to IFA supplementation was 52.7%. On the other hand, poor Hb response among anemic pregnant women with co-morbidity were high (58.8%). Particularly poor Hb response among anemia with HIV positive were 69.2%. The overall poor Hb response among anemic pregnant women was 47.3%. Residence, intestinal parasite infection, meat feeding habit, green leafy vegetables & fruit feeding habit, coffee drinking habit, parity, duration of IFA supplementation, stage of pregnancy and HBV &HCV positive were statistically significant association with poor hemoglobin responses. Hence, health care providers should be strengthened efforts to raise awareness about the importance of IFA supplementation during pregnancy and offer nutritional counseling focused on consuming an extra meal, meat, fruits, and vegetables. Longer follow-up studies starting from the first trimester are recommended to monitor supplementation outcomes and assess serum iron parameters like ferritin. Additionally, larger sample sizes and varied study designs are needed to explore potential links between factors such as abortion history and comorbidities with Hb response.

## Supporting information

S1 File Quaternary for data collection. (DOCX)
