# Supplementary material for: Hemoglobin response to iron-folic acid supplementation and associated factors among anemic pregnant women attending the University of Gondar Comprehensive Specialized Hospital ANC ward Northwest, Ethiopia 2023: A longitudinal follow up study
Source: PLoS One. 2025 Sep 4;20(9):e0331599. doi: 10.1371/journal.pone.0331599 (PMC12410736; doi:10.1371/journal.pone.0331599)
Supplement: S1 File — (DOCX) [file pone.0331599.s001.docx]

**Quaternary for data collection**

| Table 1. Socio-demographic characteristics | | | | | | | | |
| --- | --- | --- | --- | --- | --- | --- | --- | --- |
| No | Question | Response | | | | | | |
| 1 | Age |  | | | | | |  |
| 2 | Marital status | 1. Single | 2. Married | | | 3. Divorced | 4. Widowed |  |
| 3 | Educational status | 1.un educated | 2.Primary schoo | | | 3.High school | 4. Certificate and above |  |
| 4 | Occupation status | 1.Government | 2.Laborer | | | 3. Merchant | 4.others |  |
| 5 | Level of income |  |  | | |  |  |  |
| 6 | Residence | 1.Urban | 2. Rural | | |  |  |  |
| Table 2 .Obstetric factors | | | | | | | |  |
| 1 | Stage of pregnancy | 1.First trimester | 2.Second trimester | | | 3.Third trimester |  |  |
| 2 | Parity |  |  | | |  |  |  |
| 3 | ANC flow up start time |  |  | | |  |  |  |
| 4 | No of ANC visit |  |  | | |  |  |  |
| 5 | History of Abortion | Yes….. | No…… | | |  |  |  |
| Table 3.Clinical characteristics | | | | | | | |  |
| 1 | Recent illnesses | Yes……  Types….. | No…… | | |  |  |  |
| 2 | Chronic disease | Yes…..  Types… |  | | |  |  |  |
| 3 | Weekly adherence |  | |  | |  |  |  |
|  |  |  | |  | |  |  |  |
| Table 4.Nutritional characteristics | | | | | | | |  |
| 1 | Daily feeding time |  | | |  |  |  |  |
| 2 | Tea drinking | Yes | | | No |  |  |  |
| 3 | If you yes number 2 | Before meals | | | After meals | No time preference |  |  |
| 4 | Coffee drinking | Yes…… | | | No…… |  |  |  |
| 5 | If you yes number 4 | Before meals | | | After meals | No time preference |  |  |

የመረጃመጠየቂያቅፅ

መለያቁጥር__________________

| ክፍል 1: ማህበራዊሁኔታ | | |
| --- | --- | --- |
| ተ.ቁ | ጥያቄ | መልስ |
| 1 | እድሜ |  |
| 2 | የጋብቻሁኔታ | 1.ያግባች 2.ያላገባች 3. ፈት 4. በሞትየተለየ |
| 3 | የትምህርትደረጃ | 1. ያልተማች 2. አንደኛደረጃ  3.ሁለተኛደረጃ 4.ሰርትፍኬትእና ከዚያበላይ |
| 4 | የስራሁኔታ | 1.የመንግስት ሰራተኛ 2.አርሶ አደር 3. ነጋዴ 4.የቀን ሰራተኛ 5.ሌላም |
| 5 | የገቢ መጠን |  |
| 6 | የመኖሪያ ቦታ | 1.ከተማ 2.ገጠር |
| ከእርግዝና ጋር የተያያዙ ሁኔታዎች | | |
| 1 | የእርግዝና ደረጃ | 1. የመጀመሪያ ቅደድመ ወሊድ ክትትል 2. ሁለተኛ ክድመ ወሊድ ክትትል 3/ ሶስተኛ ክድመ ወሊድ ክትትል |
| 2 | ውርጃ | 1.ነበረ……. 2.አልነበረም……. |
| 3 | መድሀኒት የጀመሩበት ጊዜ |  |
| 4 | በሳምንት ስንት ጊዜ ይወስዳሉ |  |
| 5 | በህክምና የተረጋገጠ በሽታ ነበረዎት | 1.አዎ……….ምን አይነት በሽታ 2.አልነበረም………… |
| የአመጋገብ ሁኔታ | | |
| 1 | በቀን ስንትጊዜ ይመገባሉ |  |
| **2** | ሻይ ይጠጣሉ | 1.አዎ 2.አልጠጣም |
| 3 | ተ.ቁ 2. አዎ ከሆነ መልስዎ | 1. ከምግብ በፊት 2 .ከምግብ በኃላ 3. የተቀመጠ ሰአት የለም |
| 4 | ቡና ይጠጣሉ | 1.አዎ 2. አልጠጣም |
| 5 | ተ.ቁ 4 አዎ ከሆነ መልስዎ | 1.ከምግብ በፊት 2.ከምግብ በኃላ 3.የተቀመጠ ሰአት የለም |
